# Lipophilic Compounds and Antibacterial Activity of *Opuntia ficus-indica* Root Extracts from Algeria

**DOI:** 10.3390/ijms231911161

**Published:** 2022-09-22

**Authors:** Elias Benramdane, Nadia Chougui, Patrícia A. B. Ramos, Nawal Makhloufi, Abderezak Tamendjari, Armando J. D. Silvestre, Sónia A. O. Santos

**Affiliations:** 1 Département de Biologie Physico-Chimique, Faculté des Sciences de la Nature et de la Vie, Université de Bejaia, Bejaia 06000, Algeria; 2 CICECO—Aveiro Institute of Materials and Department of Chemistry, University of Aveiro, 3810-193 Aveiro, Portugal; 3 Département des Sciences Alimentaires, Faculté des Sciences de la Nature et de la Vie, Université de Bejaia, Bejaia 06000, Algeria; 4 Laboratoire de Biochimie Appliquée, Faculté des Sciences de la Nature et de la Vie, Université de Bejaia, Bejaia 06000, Algeria

**Keywords:** antibacterial activity, fatty acids, MIC, *Opuntia ficus-indica*, roots lipophilic extracts, sterols

## Abstract

The chemical composition, investigated by gas chromatography-mass spectrometry, and antibacterial activity of lipophilic extractives of three varieties of *Opuntia ficus-indica* roots from Algeria are reported in this paper for the first time. The results obtained revealed a total of 55 compounds, including fatty acids, sterols, monoglycerides and long chain aliphatic alcohols that were identified and quantified. β-Sitosterol was found as the major compound of the roots of the three varieties. Furthermore, considerable amounts of essential fatty acids (ω3, ω6, and ω9) such as oleic, linoleic, and linolenic acids were also identified. The green variety was the richest among the three studied varieties. The antibacterial activity, evaluated with disc diffusion method, revealed that lipophilic extracts were effective mainly against Gram-positive *Staphylococcus aureus* and methicillin-resistant *Staphylococcus aureus* (MRSA) (19~23 mm). Gram-negative strains mainly *Pseudomonas aeruginosa* gave an inhibition zone of 18 mm, which is considered high antibacterial activity. The minimal inhibitory concentrations of the tested bacteria revealed interesting values against the majority of bacteria tested: 75–100 µg mL^−1^ for *Bacillus* sp., 250–350 µg/mL for the two *Staphylococcus* strains, 550–600 µg mL^−1^ for *E. coli*, and 750–950 µg mL^−1^ obtained with *Pseudomonas* sp. This study allows us to conclude that the lipophilic fractions of cactus roots possess interesting phytochemicals such as steroids, some fatty acids and long chain alcohols that acted as antibiotic-like compounds countering pathogenic strains.

## 1. Introduction

Cactus plant (*Opuntia ficus-indica*) has long been used in traditional medicine for the treatment of several diseases. As a result, during the last two decades, the search for health-promoting compounds in *O. ficus-indica* became increasingly popular [1,2]. In fact, this Caryophyllal-belonging plant was revealed to be rich in a variety of bioactive secondary metabolites, namely flavonoids, betalain pigments, and tocopherols [3], which are known to play an important role in human health protection and prevention from different pathologies [4].

The attention paid to this succulent plant is in part due to its rapid growth in poor soil and low water requirement, given its complex root system in the soil and the carbon concentration mechanism in the aerial part that faces up all dryness forms [5,6]. Cactus roots also play a key role in the enrichment of soil by organic matter and so preventing it from hydraulic erosion by almost undetermined root growth [7].

Several varieties of cactus were described in the literature, namely, orange, red, green and purple [8,9] according to the colour of the fruit which is due to the accumulation in different proportions of nitrogen-containing pigments called betalains (purple betacyanins and yellow betaxanthins) [10]. Nevertheless, the green variety is exempted from these pigments [9]. In Algeria, the orange prickly pear variety is the most abundant, while the purple variety is the less available [11]. 

Several researchers have focused their work on the aerial botanical parts of cactus; the fruit (prickly pear) contains higher amounts of betalains, which are considered reference antioxidants [9,12]. Likewise, their seeds are known to be rich in fatty acids, sterols tocopherols, and glycosylated flavonols [13,14,15]. Furthermore, the cladodes and the flowers were also reported as sources of phenolic acids, glycosylated flavonols, fatty acids, sterols, vitamins and volatile compounds [8,16,17,18].

The biological activities of *O. ficus-indica* were unveiled first for antioxidant activities of the fruit extract by decreasing oxidative stress and LDL cholesterol (Low-Density Lipoprotein) in healthy humans [19]. Afterwards, gastroprotective activities were studied by Galati et al. [20], who demonstrated the proactive effect of cactus mucilage on the gastric mucosa. Furthermore, Benayad et al. reported the anti-inflammatory activity of cactus flower extracts by inhibiting the production of nitric oxide in an *in vitro* study [21]. Likewise, the same aspect is seen with a cactus pigment indicaxanthin which contributes to the anti-inflammatory activity of the Caco-2 cell line via the reduction of the expression of cyclooxygenase II (COX-II) and NO synthase enzyme in a dose-dependent manner [22]. Finally, the same pigment was described by Allegra et al. for its impairment of melanoma A375 cell line proliferation and invasiveness *in vitro* [23]. Most studies in this area were oriented to the gastrointestinal part using matching cell lines in each case [24,25]. 

Unlike the above-mentioned morphological parts, the phytochemical profile of the roots is still largely unknown, although their phenolic fraction has been evaluated for antioxidant, antiulcerogenic, and antidiabetic activities [26,27]. Hence, the aim of this study was to determine the chemical profile of *O. ficus-indica* roots, from semi-arid lands of Kabylia in north Algeria, by gas chromatography-mass spectrometry (GC-MS) analysis as well as to evaluate the bacterial impairment of their lipophilic fractions, namely against *Escherichia coli*, *Pseudomonas aeruginosa*, *Staphylococcus aureus* and one of its methicillin-resistant strain, and finally *Bacillus cereus*, strains that cause health disorders via food poisoning, gut microbiota disorder [28], soft tissues, bloodstream and urinary tract infections [29], as well as pneumonia leading to death in several cases [30].

## 2. Results and Discussion

### 2.1. Physicochemical Properties of O. ficus-indica Roots

The results of the physicochemical properties of fresh roots are presented in Table 1. The moisture content was found in the range of 76–81% in all the varieties. These values are lower than those found for the stems (≥90%) [31]. This parameter is directly related both to the soil moisture and the metabolism of the plant [7,32]. The rate of titratable acidity was found to be higher in the orange variety (0.26 ± 0.01%), with significant differences noticed between the three varieties. This value is higher than that found in the fruits (0.058%) [33] and falls in the same range found for the stems [31]. The pH values were registered between 5 and 7. The orange variety—which presented the highest acidity rate—showed the lowest pH index (5.25 ± 0.19), with the same trend observed for the red variety, which presented the lowest acidity rate (pH = 6.16 ± 0.09). The green variety contains the highest amount of total soluble solids (Brix) with a value of 10.75% compared to the red and orange varieties that present the lowest percentages. These values are higher than those reported by Nabil et al. [34] for the cladode powder and fall in the same range as the brix values of the fruit [35]. These values represent considerable amounts of complex sugars that contribute to the total soluble solids (TSS) of the roots, which enhance the quality of the soil with organic matter [36].

### 2.2. Lipophilic Fraction of O. ficus-indica Roots

#### 2.2.1. Extraction Yield

The dichloromethane (DCM) extracts from *O. ficus-indica* roots presented significantly different extraction yields. The green variety gave the highest amount of lipophilics with 5.78 ± 0.01 g kg^−1^ dw, followed by the orange variety with 4.74 ± 0.03 g kg^−1^ dw, while the red cultivar showed the lowest yield with 2.72 ± 0.03 g kg^−1^ dw. Being harvested in the same location and climatic conditions, these differences are mainly due to the difference in varieties and are also caused by the edaphic conditions and the shallowness of the roots. Furthermore, the root extraction yield is constantly lower than that of the aerial parts [37]. Furthermore, no data about cactus root extraction yields have been reported so far in the literature. However, the values reported here are higher than those reported for cladodes (1.2 g kg^−1^) [38] but lower than those of cactus fruit peels (36.8 g kg^−1^) [39]. Angulo-Bejarano et al. reported a value of 2 g kg^−1^ of lipids in the fresh cactus stem, which is closer to the yield value of the red variety in our study [40]. Finally, the contents reported in this study are similar to those of the fruit seed found by Taoufik et al. for diameters of seeds in the range of 1.25 and 1.80 mm [14].

#### 2.2.2. Lipophilic Composition

**Figure 1:**
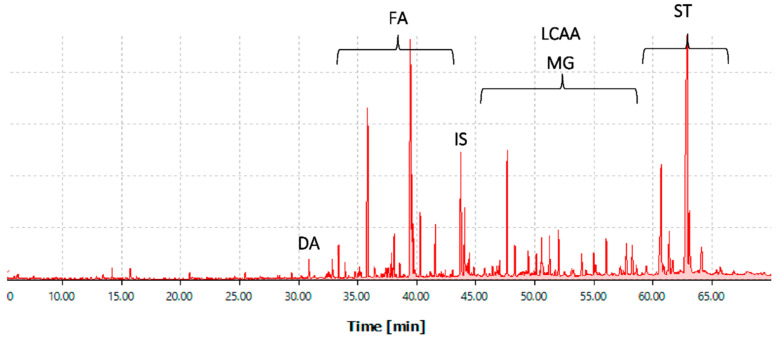
GC–MS chromatogram of trimethylsilylated DCM extract of *Opuntia ficus-indica* harvested in Kabylia area. Abbreviations: DA, dicarboxylic acids; FA, fatty acids; IS, internal standard; LCAA, long chain aliphatic alcohols; MG, monoglycerides; ST, sterols.

**Figure 2:**
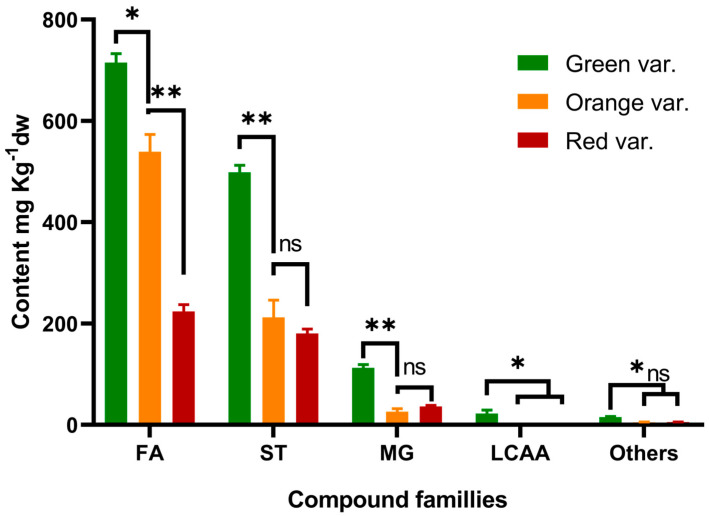
Major families of lipophilic compounds identified in dichloromethane extracts of three varieties of *Opuntia ficus-indica* roots. *, significant difference at *p* ≤ 0.05; **, significant difference at *p* ≤ 0.01; ns, non-significant difference.

The chemical composition of the DCM extracts of the three root varieties of *O. ficus-indica* that was investigated by GC-MS analyses is summarized in Table 2. Four main families of lipophilic compounds were identified and quantified, namely fatty acids, long chain aliphatic alcohols, sterols, monoglycerides and other minor metabolites as illustrated in Figure 1 and Figure 2 and Table 2. 53 compounds were identified in the green variety, while 37 was found in the orange and 36 in the red varieties.

The total amount of lipophilic compounds quantified in the roots of *O. ficus-indica* was 1363 mg kg^−1^ in the green variety, 783 mg kg^−1^ in the orange and 446 mg kg^−1^ in the red one.

Considering the main families of lipophilic compounds (Table 2 and Figure 2) fatty acids were the main family present in the three varieties with content values of 715, 539 and 224 mg kg^−1^ dw, representing rates of 52.5%, 68.8%, and 50.2% of the total lipophilic compounds respectively for green, orange, and red varieties. Furthermore, sterols were found as a second major family accounting for 499, 212, and 180 mg kg^−1^ dw, respectively. Smaller amounts of monoglycerides (from 3.3% to 8.2%) were quantified in the three varieties with amounts ranging between 26 and 112 mg kg^−1^ dw, while long chain aliphatic alcohols were found only in the green variety with an amount of 22 mg kg^−1^ dw representing about 1.6% of the total lipophilic compounds detected. All the compounds reported in this study were identified for the first time in *O. ficus-indica* roots.

##### Fatty Acids

Unsaturated fatty acids (UFAs)

Thirteen unsaturated fatty acids (C_15_–C_20_) were detected in the DCM extracts of *O. ficus-indica* roots (Table 2). Their content varied significantly from 94 mg kg^−1^ dw in the red variety to 358 mg kg^−1^ dw in the orange one. The three major compounds identified were oleic (ω9), linoleic (ω6), and linolenic (ω3) acids, with contents significantly ranging from 14 to 93 mg kg^−1^ dw, from 21 to 213 mg kg^−1^ dw, and from 6 to 38 mg kg^−1^ dw for the three compounds, respectively. The green variety was the richest in terms of UFAs, followed by the orange and the red ones. The two former fatty acids were reported in our previous study as major compounds in Algerian cactus seed oils of orange and red varieties with much higher concentrations [13]. (9E)-Octadec-9-enoic and nonadecadienoic acids showed relevant amounts mainly in the green variety, with values of 16 mg kg^−1^ and 19 mg kg^−1^ dw, respectively. UFAs are considered essential and important in the human diet since they are not synthesized by the organism. The intake of these compounds improves cognitive function and behaviour, decelerates the inflammatory process and prevents cardiovascular diseases, diabetes and cancer [41].

Saturated fatty acids (SFAs)

SFAs were found in relatively higher amounts in the characterized samples, mainly within orange and red variety extracts. Fifteen SFAs were detected in the three fractions, with higher contents in the orange and green varieties. The content of SFAs in the roots significantly ranged from 129 mg kg^−1^ dw in the red variety to 354 mg kg^−1^ dw in the green one. Being most abundant in plant lipophilic compounds, palmitic acid (C_16_) was the major fatty acid found in our samples with contents ranging from 59 mg kg^−1^ dw in the red variety to 183 mg kg^−1^ dw in the orange one. Stearic (C_18_), lignoceric (C_24_) and cerotic (C_26_) acids were found in a descending order mainly with higher amounts in the green variety; amounts of 31, 23, and 22 mg kg^−1^ dw were noticed respectively for the three compounds. This class of fatty acids are also known to play a key role in preventing cardiovascular diseases, and mainly those related to coronary heart disease [42,43]. They are also potent antibacterial agents against different pathogen strains [44,45].

**Figure 3:**
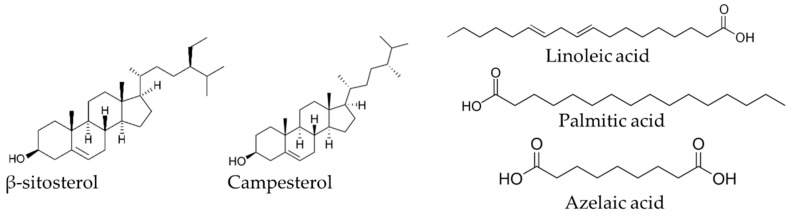
Major lipophilic compounds detected in Algerian *Opuntia ficus-indica* root extracts.

Finally, a diacid acid, namely, azelaic acid was also found in lipophilic extracts (Figure 3), with a content of 46 mg kg^−1^ dw in the orange variety and with smaller amounts found in green (3 mg kg^−1^ dw) and red (1 mg kg^−1^ dw) ones.

##### Long-Chain Aliphatic Alcohols

Long-chain aliphatic alcohols were only found in the green variety. Seven compounds were detected in contents that varied from 2 to 6 mg kg^−1^ dw. Triacontan-1-ol was the major long-chain aliphatic alcohol found with an amount of 6 mg kg^−1^ dw, followed by hexadecan-1-ol and octadecane-1-ol both with contents of 4 mg kg^−1^ dw. This class of lipophilic compounds has been demonstrated to present antibacterial activity mainly against *Staphylococcus* spp. [46], *Streptococcus* spp. [47], and *Mycobacterium* spp. [48]. 

##### Sterols

β-Sitosterol was the major sterol identified among the eleven steroids found in the three studied varieties, with amounts ranging from 130 mg kg^−1^ dw in the red variety to 352 mg kg^−1^ in the green one (*p* < 0.05) (Table 2). Campesterol (with contents from 26 in red variety to 84 mg kg^−1^ dw in the green one) and stigmastanol (from 12 in the red variety to 38 mg kg^−1^ in the green one) were, respectively, the second and the third sterols identified in all the extracts. Finally, stigmasterol was found at the lowest concentration, and the highest was mainly in the green variety (25 mg kg^−1^ dw). β -sitosterol was already reported as a the major sterol identified in the cladodes and in the fruit skin of *O. ficus-indica* in a semi-quantitative study but also in the seed oil with a rate of 61.42% [49,50,51]. Campesterol was also identified as a second major compound, with a rate of 16.55% in the same oil, and with a rate of 11.04% in another study [52]. All sterols reported here were identified for the first time in *O. ficus-indica* roots. Steroid-like compounds are known to exhibit some physiological benefit activities such as reduction of the plasma cholesterol level [53,54], anti-inflammatory and antitumor activities [55,56], as well as bacteria inhibition, mainly against *S. aureus*, *E. coli*, *Salmonella* sp. and *Klebsiella* sp. [57,58].

##### Monoglycerides

Twelve monoglycerides were identified in the root extracts of *O. ficus-indica*. Their content varied significantly from 26 mg kg^−1^ dw in the orange variety to 112 mg kg^−1^ dw in the green one. 2,3-Dihydroxypropyl hexadecanoate (1-monopalmitin) was the major compound detected with a concentration of 49 mg kg^−1^ dw in the green variety. 2,3- Dihydroxypropyl octadecenoate (monostearin) and 2,3-dihydroxypropyl (9Z,12Z)-octadeca-9,12-dienoate (monolinolein) came, respectively, in the second and the third place with amounts of 14 and 12 mg kg^−1^ dw within the same variety. However, lower amounts were detected in the other two varieties. Within this class of compounds, the red variety was revealed to be more affluent in comparison with the orange one. The remaining monoglycerides were under 10 mg kg^−1^ dw. Some studies reported the antimicrobial activities of this class of compounds against Gram-negative pathogens essentially [59] and evenly against fungi such as *Fusarium* spp., *Aspergillus* spp. and *Penicillium* spp. [60,61].

##### Other Compounds Identified

Other compounds were detected at smaller amounts: glycerol and α-tocopherol were found at values of 6 and 9 mg kg^−1^ dw in the green variety, respectively. The first compound presented content of 4 mg kg^−1^ dw in the orange variety, whereas α-tocopherol was found at the same amount in the red variety. Finally, (9E)-octadec-9-enoic acid ethyl ester was detected in the orange variety at an amount of 1 mg kg^−1^ dw. α-Tocopherol which represents the active form of vitamin E is known for its health benefits mainly against reactive oxygen species preventing oxidative and inflammatory damage as well as in the prevention of some dysfunction pathologies like diabetes and vasculopathies [62].

### 2.3. Antibacterial Activity

#### 2.3.1. Bacteria Inhibition on Agar Medium

**Figure 4:**
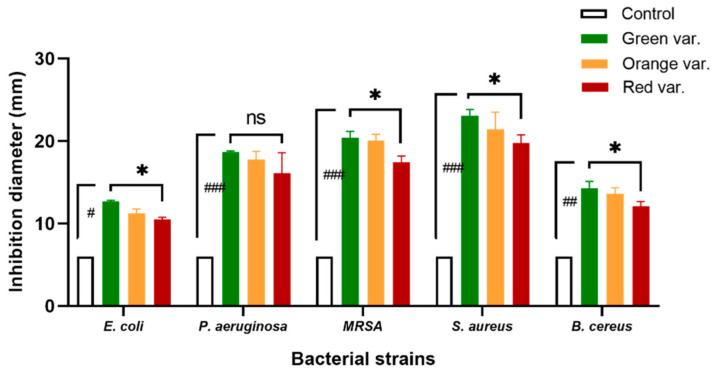
Histograms of inhibition zones obtained with 3 varieties of cactus root extracts. #: weak antibacterial activity, ##: moderate antibacterial activity, ###: high antibacterial activity [66], ns: none significative, *: significant difference (*p* = 0.05) between the three varieties using Tukey’s HSD test.

In this preliminary assay, broader spectrum bacterial inhibition was accessed for the extracts of the three *O. ficus-indica* root varieties. The largest inhibition diameters were noticed against *S. aureus* sp., whereas the smallest zones were obtained against *E. coli* strain. Among the three varieties, the green one showed the highest bacteria inhibition vis-a-vis all the strains (Figure 4). In a global view, the Gram-positive class was more sensitive to the lipophilic extracts used than the Gram-negative one, evidence that was already reported in several studies [63,64,65]. To the best of our knowledge, no antibacterial activity related to the cactus root lipophilic extracts has been reported so far.

With regard to the Gram-positive strains, the green variety extract showed inhibition zones of 23.08 mm and 20.42 mm against *S. aureus* ATCC 29213 and *MRSA*, respectively. *B. cereus* was less sensitive to this same extract (14.25 mm diameter). Orange variety extract activity was in the same trend against these strains with a slightly shorter diameter compared to the green cultivar (*p* > 0.05). Downhill, extract from the red variety was less effective in countering the same strains; zone diameters of 12.08 mm, 17.42 mm, and 19.75 mm were recorded for *Bacillus cereus*, MRSA, and *S. aureus*, respectively. An anterior study confirmed the same tendency of bacteria sensitivity using the ethanol extract of cactus fruit peels with smaller diameters; 15.00 mm for both *S. aureus* and *MRSA*, and 11.30 mm for *B. cereus* [67]. In the same optic, another study reported the inhibition effect of the unsaponifiable fraction of cactus oil with a diameter of 12.70 mm at 100 µg mL^−1^ against *S. aureus* ATCC 25923 [68]. In previously reported studies, the same strain was tested using the hexane extract of Tunisian cactus flowers at two flowering stages, and the optimum activity was registered at the full-flowering stage, with a diameter of 15.70 mm [69]. Additionally, the ethyl-acetate extract of the cactus fruit peel was demonstrated to be effective in the same range towards *S. aureus* (15.14 mm) and a lower activity against *B. cereus* (9.68 mm) [51]. Finally, for the latter, the same range inhibition was recorded with the chloroform extract of the cactus stem against *B. subtilis* (10.23 mm) [70] which is significantly lower than our results.

The two Gram-negative strains, on the other hand, were found to be less sensitive to the lipophilic extracts under study. *P. aeruginosa* was inhibited effectively by the green extract (18.67 mm), as though the extracts of the orange and red varieties acted likewise, giving inhibition zones in the same range with 17.75 and 16.08 mm, respectively. No significant differences were registered in this case. *E. coli* strain was found as the most resistant to the lipophilic extracts under study with the smallest inhibition zones; 12.67, 11.25, and 10.50 mm with the green, orange, and red varieties, respectively, which are nonetheless considered moderate antimicrobial activities according to Vaquero et al. [66]. Significant differences were observed between the red and the green varieties in the case of *E. coli*. Our results are in agreement with those previously reported by Ennouri et al. [69] who registered 18.80 ± 0.80 mm diameter with the hexane extract but at 100 mg/mL of cactus flowers against *P. aeruginosa*. However, 8.50 ± 0.80 mm inhibition was obtained at the same concentration by R’bia et al., with the unsaponifiable fraction of cactus seed oil against the same strain [68]. Furthermore, no inhibition zone was observed against *P. aeruginosa* with the ethyl acetate fruit extract reported by Bargougui et al., unlike *E. coli* strain, which was sensitive, giving 10.00 ± 0.56 mm diameter with one Algerian variety [71]. This finding is in the same range that was achieved for this specie in our study. Ortega-Ortega et al. found an inhibition zone of 7.56 ± 0.19 mm with cactus seed oil extracted by hexane [72], whereas 13.00 ± 2.2 mm were obtained by R’bia et al. with the unsaponifiable fraction of the same oil [68], which is evenly in the same range as found in our study. Finally, similar zones diameters were obtained by El-Beltagi et al. using the ethyl acetate extracts of the pulp and the peels of prickly pears (10.32 ± 0.10 mm and 11.17 ± 0.18 mm against *E. coli*, respectively) [51].

#### 2.3.2. Minimal Inhibitory Concentration

The minimal inhibitory concentrations (MICs) that inhibit the growth of the five tested strains are presented in Table 3. The most effective and lowest concentrations were obtained against the Gram-positive class. The MIC against *B. cereus* was inhibited at 76.67 ± 5.77 µg mL^−1^ with the green variety extract; the orange and red extracts gave MICs in the same range (83.33 ± 5.77 and 86.67 ± 5.77 µg mL^−1^) (no statistical difference). Both referenced *S. aureus* and *MRSA* had a closer sensitivity to the green and orange varieties, whereas, to the red variety, a 1.3-fold difference was noticed compared to these two strains. 

As previously reported, the lipophilic extracts tend to be more effective in terms of bacteria inhibition than the hydrophilic ones. Mabotja et al. stated MICs of several cactus varieties using the methanol and the petroleum ether extracts of cladodes, in most cases, the petroleum ether extract was 10-fold more effective [73]. *Bacillus* sp. was inhibited at 0.39 mg mL^−1^, while *S. aureus* was at 0.78 mg mL^−1^ in the same study. A similar trend was observed against *B. cereus* and *S. aureus* with the hexane extract of cactus fruit peels (5.00 and 2.50 mg mL^−1^, respectively), while the concentrations of the acidified methanol fraction were higher (6.25 mg mL^−1^ and 9.38 mg mL^−1^, respectively) [67].

Within the Gram-negative class, the less effective MIC was recorded against *P. aeruginosa* at 947.50 ± 5.00 µg mL^−1^ with the red extract, whereas, *E. coli* was found more sensitive within the liquid medium, with a MIC of 550.00 ± 0.00 µg mL^−1^ estimated for the green variety, while higher MICs, namely 566.67 ± 5.77 µg mL^−1^ and 606.00 ± 5.48 µg mL^−1^ were noticed with the orange and the red varieties, respectively (*p* < 0.05). This class of bacteria is known to have an internal membrane that protects the cell from external aggressions, which may explain the high concentrations used in this study comparing those used against the Gram positives. Our results are in agreement with those found by Mabotja et al. which inhibited *E. coli* strain by petroleum ether lipophilic fraction with concentrations varied between 0.39 and 0.78 mg mL^−1^ of cactus pear extracts [73]. Karadağ et al. revealed inhibition concentrations superior to 1000 µg mL^−1^ testing *P. aeruginosa* and *E. coli* sensitivity towards hexane extracts of cactus fruit [74]. In another different study, carried out by Blando et al., the polyphenolic extract of cactus cladodes inhibited *E. coli* strain at a concentration 3-fold higher than that indicated in our study (1500 µg mL^−1^) [75]. 

The foremost compounds acting as antibacterial agents in our extracts are expected to be sterols and long-chain aliphatic alcohols. These two classes of bioactive compounds were reported elsewhere to have antimicrobial effects [46,76,77]. β-sitosterol, stigmasterol and campesterol were effective against *E. coli*, *P. aeruginosa*, *S. aureus*, and *Bacillus* sp. [76,78]. 1-octacosanol and 1-pentacosanol were likewise reported to act as bacterial inhibitors against the same strains according to Feng et al. [77]. This last author reported the antibacterial effect of octacosanoic acid evenly, a saturated fatty acid which is identified in this study. The synergic effect of different compounds contained in these extracts is eventually evoked by analysing the MICs obtained in this study mainly against *B. cereus* and *E. coli*, a hypothesis that is always put forward in such studies [69,79].

## 3. Materials and Methods

### 3.1. Reagents

Dichloromethane (p.a., ≥99% purity) was purchased from Fisher Scientific (Thermo Fisher Scientific, Waltham, MS, USA). Anhydrous pyridine (99.8% purity), *N,O*-bis(trimethylsilyl)-trifluoroacetamide (99% purity), trimethylchlorosilane (99% purity), tetracosane (≥99% purity), pentadecan-1-ol (99% purity), hexadecanoic acid (≥99% purity), *β*-stigmasterol (95% purity) and vanillin (99% purity), were supplied by Sigma Chemical^©^ (Madrid). Dimethyl sulfoxide (DMSO) was supplied from Sigma Aldrich^©^ (Darmstadt, Germany), BHIB and Mueller-Hinton mediums were provided by BIOKAR^©^ (BIOKAR Diagnostics, Allonne, France).

### 3.2. Harvest and Post-Harvest Processes

Three varieties, namely orange, green, and red of *Opuntia ficus-indica,* were harvested in August 2018. The orange variety was collected from the region of El-Kseur, at 30 km from Bejaia city, Algeria (36°41′28.11″ N 4°49′01.82″ E), whereas the green and the red varieties were taken from At Wasif at 35 km in the south of Tizi-Ouzou city in north Algeria (36°32′27.18″ N 4°12′00.15″ E; 36°31′43.25″ N/4°10′56.20″ E), an area which is characterised by a semi-arid climate with hot and dry summers and somewhat cold and rainy winters, with temperatures ranging between 23 and 45 °C. Roots were transported to the laboratory and immediately washed with distilled water, sliced into small cubes and crushed using an electric grinder in order to determine the physicochemical parameters. The fractions destined for solvent extraction were freeze-dried, ground to a fine powder (ϕ ≤ 125 µm) and stored at room temperature in sealed containers until use. 

### 3.3. Physicochemical Proprieties Measurement

Physicochemical parameters were determined by conventional methods: moisture was measured at 105 °C according to AOAC 1990 [80], titratable acidity, expressed as the percentage of citric acid, and pH was evaluated in the juice matrix, as reported by El Kharrassi et al. [35], and the brix percentage was measured using a refractometer.

### 3.4. Extraction of Lipophilic Compounds

Lipophilic compounds were extracted using a Soxhlet apparatus: 10 g of root powder was extracted using 180 mL of dichloromethane (DCM) for 8 h according to Ramos et al. [81]. Next, the solvent was evaporated completely at 40 °C at low pressure using Büchi R-200 rotavapor (Büchi, Flawil, Switzerland) and the remaining dry residue was weighed and expressed as % of dry weight (dw). Extracts were obtained in triplicate.

### 3.5. Gas Chromatography-Mass Spectrometry Analysis

Dichloromethane extracts were analysed by GC-MS. Beforehand, the samples were subjected to derivatization by trimethylsilylation. Briefly, each sample was dissolved in 250 µL of pyridine containing 0.6 mg of tetracosane as internal standard (IS), after that 250 µL of *N,O*-bis(trimethylsilyl)-trifluoroacetamide and 50 µL of trimethylchlorosilane were added and the mixture was incubated at 70 °C for 30 min in an oil bath [82,83]. 

The derivatized extracts were analysed by GC–MS using a QP2010 Ultra (Shimadzu, Kyoto, Japan). Compounds were separated in a DB-1 J&W capillary column (30 m × 0.32 mm inner diameter, 0.25 µm film thickness, Santa Clara, CA, USA), using helium as the carrier gas (35 cm s^−1^). The temperature program was as follows: initial temperature, 80 °C for 5 min; temperature rate, 4 °C min^−1^ up to 260 °C; temperature rate, 2 °C min^−1^ up to 285 °C which was kept for 8 min. The injector and the transfer-line temperatures were, respectively, at 250 °C and 290 °C, while the split ratio was 1:33. The mass spectrometer was operated in the electron impact mode at 70 eV, and the data were collected at a rate of 1 scan per second over a range of *m*/*z* 33–700. The ion source was maintained at 250 °C [84].

Compounds were identified by comparing their spectra with the GC–MS spectral library (Wiley-NIST Mass Spectral Library 2014) and with the published data [81,84,85,86,87], and in some cases by injection of standards. 

Quantification was done based on the internal standard peak area. In addition, response factors of the different families of compounds in relation to tetracosane were determined using reference standards, representative of the different families of compounds detected, namely hexadecanoic acid (fatty acids), pentadecan-1-ol (long chain aliphatic alcohols), stigmasterol (sterols), and vanillin (aromatic compounds). Response factors were determined from the mean of six GC-MS runs. For each variety, three derivatized extracts were prepared, and each one was injected in duplicate (*n* = 6).

### 3.6. Antibacterial Activity

#### 3.6.1. Bacterial Strains and Culture Conditions

The Gram-negative strains *Escherichia coli* (ATCC 25922) and *Pseudomonas aeruginosa* (ATCC 27853) and the Gram-positive *Staphylococcus aureus* (ATCC 29213), Methicillin Resistant *Staphylococcus aureus* (*MRSA*) ref. MU45 (*Mec C*), and *Bacillus cereus* (ATCC 10876) were used in the present study.

The strains were planted out in the brain-heart infusion broth (BHIB) at 37 °C for 24 h. Afterwards, the bacterial strains were cultivated at the same temperature for 12 h in Mueller–Hinton agar medium. In order to work with fresh bacteria, the BHIB was used again with each strain for 18 h and then the standardisation of the bacterial suspension was made by measuring optical density at 600 nm using a UV-visible spectrophotometer (SECOMAM, Alès, France) after dilution of the bacterial suspension with sterile PBS (pH = 7.4). Absorbances between 0.08 and 0.1 were obtained for 1–2 × 10^8^ colony-forming units mL^−1^. The stock solutions were conserved in a cryoprotection medium at −80 °C. Revivification in BHIB was made before each test.

#### 3.6.2. Agar Medium Diffusion Test

The evaluation of the antibacterial activity of the lipophilic root extracts of *O. ficus-indica* was determined following the standard protocol published by SFM 2019 [88]. Petri dishes containing 4-millimetre Mueller–Hinton agar medium were used for this purpose. 100 mL of the standardized bacteria inoculum of each strain was experienced in the Petri dishes at room temperature and in sterile conditions. After spreading out uniformly the bacteria using a medical swab, sterile discs of 6 mm diameter were placed on the agar medium and impregnated with 10 µL of each extract. The dishes were left for a maximum period of 15 min before incubation at 37 °C for 24 h. The antibacterial activity was evaluated by measuring the inhibition zone (in mm) around the paper discs using a calliper. Each assay was experimented with in triplicate.

#### 3.6.3. Determination of the Minimal Inhibition Concentration (MIC)

The MICs of the tested extracts were determined against the targeted bacterial strains. As used for the diffusion test, fresh standardized bacterial inoculum (5 µL) was loaded in a 96-well microplate using Mueller-Hinton broth as a culture medium (95 µL). After the preparation of the dilution series of the extracts in DMSO, 100 µL of each extract concentration was added to a final volume of 200 µL. The incubation was done at 37 °C for 24 h. The MIC was read in triplicate as the first well where no trouble is seen (no bacterial growth) [89].

### 3.7. Statistical Analysis

Triplicate-performed tests were averaged and presented as means ± SD. The variance analyses were performed by Tukey’s HSD post-hoc ANOVA test using Statistica 7.1 (Statsoft^®^, Hamburg, Germany). The graphs were plotted with the use of GraphPad Prism 8.0.1 Software (San Diego, CA, USA). In all cases, *p* = 0.05 was fixed as a significative threshold.

## 4. Conclusions

This study allowed us to know in detail the lipophilic composition of cactus roots cultivated in Algeria. The GC-MS analyses pointed out promising compounds such as β-sitosterol, stigmasterol and campesterol for steroid-like compounds, octacosanoic and linoleic acids for fatty acids and octacosanol with regard to long-chain alcohols. All these compounds could be exploited as therapeutic agents in the clinical domain against pathogenic strains but also in cosmetic applications. It is important to remind that this part of the cactus is in an almost undeterminable growth which makes it an everlasting source of bioactive compounds. Moreover, the broader antibacterial activity of the crude extracts with low MICs (3 to 5-fold lower) led to consider cactus roots as an eventual natural source of agents against pathogens. Finally, further studies would be of interest mainly to exploit sustainable extraction methodologies that will allow the exploitation of *O. ficus-indica* as a source of antibacterial agents.

## Figures and Tables

**Table 1:** Physicochemical characteristics of *Opuntia ficus-indica* roots.

	Green	Red	Orange
**Moisture (%)**	78.71 ± 0.16 ^b^ *	80.87 ± 0.14 ^a^	76.99 ± 0.08 ^c^
**Acidity (% citric acid)**	0.24 ± 0.01 ^b^	0.16 ± 0.01 ^c^	0.26 ± 0.01 ^a^
**Brix (%)**	10.75 ± 0.00	8.75 ± 0.00	7.00 ± 0.00
**pH**	5.49 ± 0.26 ^b^	6.16 ± 0.09 ^a^	5.25 ± 0.19 ^b^

* Tukey’s HSD test; difference in the letter in a line indicates a significant difference at *p* ≤ 0.05 between the varieties.

**Table 2:** Chemical composition of root lipophilic fractions from three varieties of *Opuntia ficus-indica* cultivated in Algeria.

		Content mg g^−1^ Extract			Content mg kg^−1^ dw		
RT (min)	Compound Name	Green var.	Orange var.	Red var.	Green var.	Orange var.	Red var.
	Fatty acids	123.75 ^a^ *	115.74 ^a^	89.35 ^b^	715 ^a^	539 ^b^	224 ^c^
	Saturated fatty acids	61.32 ^b^	71.55 ^a^	51.85 ^c^	354 ^a^	349 ^a^	129 ^b^
30.86	Tetradecanoic acid	1.17 ^b^	1.03 ^c^	2.04 ^a^	7 ^a^	5 ^b^	6 ^b^
33.37	Pentadecanoic acid	1.80 ^b^	2.23 ^a^	0.97 ^c^	10 ^b^	11 ^a^	3 ^c^
35.86	Hexadecanoic acid	29.51 ^b^	36.86 ^a^	24.46 ^c^	170 ^a^	183 ^a^	59 ^b^
38.08	Heptadecanoic acid	2.80 ^b^	3.90 ^a^	1.37 ^c^	16 ^b^	20 ^a^	3 ^c^
40.31	Octadecanoic acid	5.43 ^b^	7.16 ^a^	3.88 ^c^	31 ^b^	38 ^a^	8 ^c^
42.40	Nonadecanoic acid	0.40 ^b^	0.70 ^a^	ND ^c^	2 ^b^	4 ^a^	ND ^c^
44.44	Eicosanoic acid	1.37 ^b^	1.99 ^a^	1.04 ^c^	8 ^b^	9 ^a^	2 ^c^
46.41	Heneicosanoic acid	0.56 ^b^	0.79 ^a^	0.39 ^c^	3 ^b^	4 ^a^	1 ^c^
48.29	Docosanoic acid	1.93 ^a^	1.54 ^b^	1.26 ^c^	10 ^a^	8 ^b^	3 ^c^
50.12	Tricosanoic acid	1.55 ^b^	1.72 ^a^	1.36 ^c^	9 ^a^	8 ^a^	3 ^b^
52.00	Tetracosanoic acid	4.05 ^a^	2.61 ^c^	3.18 ^b^	23 ^a^	10 ^b^	8 ^c^
53.97	Pentacosanoic acid	2.09 ^a^	1.81 ^a^	1.89 ^a^	12 ^a^	6 ^b^	5 ^b^
56.04	Hexacosanoic acid	3.75 ^a^	4.29 ^a^	3.71 ^a^	22 ^a^	21 ^a^	10 ^b^
58.35	Heptacosanoic acid	3.07 ^a^	1.97 ^b^	2.98 ^a^	18 ^a^	9 ^b^	8 ^b^
60.51	Octacosanoic acid	1.83 ^b^	2.95 ^a^	3.32 ^a^	12 ^ab^	14 ^a^	9 ^b^
	Unsaturated fatty acids	61.88 ^a^	32.20 ^b^	37.08 ^b^	358 ^a^	144 ^b^	94 ^c^
32.84	Pentadecenoic acid	1.43 ^a^	ND ^b^	1.44 ^a^	8 ^a^	ND ^c^	4 ^b^
34.99	(9Z)-Hexadec-9-enoic acid	0.32 ^a^	0.20 ^b^	0.29 ^a^	2 ^a^	1 ^b^	1 ^b^
35.11	(9E)-Hexadec-9-enoic acid	0.63 ^a^	0.76 ^a^	ND ^b^	3 ^a^	3 ^a^	ND ^b^
37.37	(10Z)-Heptadec-10-enoic acid	0.57 ^a^	0.80 ^a^	ND ^b^	3 ^a^	4 ^a^	ND ^b^
37.53	(10E)-Heptadec-10-enoic acid	1.03 ^a^	1.26 ^a^	ND ^b^	5 ^a^	3 ^b^	ND ^c^
39.35	(9Z, 12Z)-Octadeca-9,12-dienoic acid	36.62 ^a^	4.76 ^c^	24.67 ^b^	213 ^a^	21 ^c^	60 ^b^
39.43	(9Z, 12Z, 15Z)-Octadeca-9,12,15-trienoic acid	6.49 ^a^	ND ^c^	2.51 ^b^	38 ^a^	ND ^c^	6 ^b^
39.53	(9Z)-Octadec-9-enoic acid	7.94 ^b^	20.40 ^a^	5.49 ^b^	45 ^b^	93 ^a^	14 ^c^
39.67	(9E)-Octadec-9-enoic acid	2.49 ^b^	4.02 ^a^	1.19 ^c^	16 ^a^	18 ^a^	4 ^b^
39.8	Octadecenoic acid isomer	0.42 ^a^	ND ^b^	ND ^b^	2 ^a^	ND ^b^	ND ^b^
41.51	Nonadecadienoic acid isomer	3.26 ^a^	ND ^c^	1.49 ^b^	19 ^a^	ND ^c^	4 ^b^
42.01	Nonadecenoic acid isomer	0.32 ^a^	ND ^b^	ND ^b^	2 ^a^	ND ^b^	ND ^b^
43.84	Eicosenoic acid isomer	0.35 ^a^	ND ^b^	ND ^b^	2 ^a^	ND ^b^	ND ^b^
	Diacids	0.54 ^b^	11.99 ^a^	0.42 ^b^	3 ^b^	46 ^a^	1 ^b^
29.36	Azelaic acid (nonanedioic acid)	0.54 ^b^	11.99 ^a^	0.42 ^b^	3 ^b^	46 ^a^	1 ^b^
	Long chain aliphatic alcohols	2.82 ^a^	ND ^b^	ND ^b^	22 ^a^	ND ^b^	ND ^b^
33.92	Hexadecan-1-ol	0.11 ^a^	ND ^b^	ND ^b^	4 ^a^	ND ^b^	ND ^b^
38.56	Octadecan-1-ol	0.06 ^a^	ND ^b^	ND ^b^	4 ^a^	ND ^b^	ND ^b^
46.78	Docosan-1-ol	0.52 ^a^	ND ^b^	ND ^b^	3 ^a^	ND ^b^	ND ^b^
50.46	Tetracosan-1-ol	0.32 ^a^	ND ^b^	ND ^b^	2 ^a^	ND ^b^	ND ^b^
54.31	Hexacosan-1-ol	0.37 ^a^	ND ^b^	ND ^b^	2 ^a^	ND ^b^	ND ^b^
58.58	Octacosan-1-ol	0.35 ^a^	ND ^b^	ND ^b^	2 ^a^	ND ^b^	ND ^b^
63.16	Triacontan-1-ol	1.09 ^a^	ND ^b^	ND ^b^	6 ^a^	ND ^b^	ND ^b^
	Sterols	86.26 ^a^	49.61 ^b^	66.78 ^ab^	499 ^a^	212 ^b^	180 ^b^
60.73	Campesterol	14.53 ^a^	6.29 ^c^	9.95 ^b^	84 ^a^	28 ^b^	26 ^b^
61.35	Stigmasterol	4.33 ^a^	0.39 ^b^	4.35 ^a^	25 ^a^	2 ^c^	12 ^b^
62.65	β-Sitosterol	60.88 ^a^	34.91 ^b^	47.23 ^ab^	352 ^a^	158 ^b^	130 ^b^
62.81	Stigmastanol	6.53 ^ab^	8.02 ^a^	5.25 ^b^	38 ^a^	24 ^b^	12 ^c^
	Monoglycerides	19.04 ^a^	4.27 ^c^	14.00 ^b^	112 ^a^	26 ^c^	36 ^b^
45.77	2,3-Dihydroxypropyl pentadecanoate	0.63 ^a^	ND ^b^	ND ^b^	3 ^a^	ND ^b^	ND ^b^
47.00	1,3-Dihydroxypropan-2-yl hexadecanoate	0.84 ^a^	0.10 ^c^	0.42 ^b^	5 ^a^	1 ^b^	1 ^b^
47.68	2,3-Dihydroxypropyl hexadecanoate	8.56 ^a^	1.99 ^c^	4.86 ^b^	49 ^a^	10 ^c^	12 ^b^
49.44	2,3-Dihydroxypropyl heptadecanoate	1.30 ^a^	0.58 ^b^	0.62 ^b^	9 ^a^	3 ^b^	2 ^b^
49.92	1,3-Dihydroxypropan-2-yl (9Z,12Z)-octadeca-9,12-dienoate	0.28 ^b^	ND ^c^	0.43 ^a^	2 ^a^	ND ^c^	1 ^b^
50.56	2,3-Dihydroxypropyl (9Z,12Z)-octadeca-9,12-dienoate	2.24 ^b^	0.10 ^c^	4.99 ^a^	12 ^a^	5 ^b^	12 ^a^
50.59	2,3-Dihydroxypropyl (9Z)-octadec-9-enoate	0.62 ^a^	0.26 ^b^	ND ^c^	5 ^a^	1 ^b^	ND ^c^
50.67	2,3-Dihydroxypropyl (9Z)-octadec-9-enoate	ND ^b^	ND ^b^	0.97 ^a^	ND ^b^	ND ^b^	3 ^a^
51.23	2,3-Dihydroxypropyl octadecanoate	2.44 ^a^	0.71 ^c^	1.29 ^b^	14 ^a^	3 ^b^	3 ^b^
53.11	2,3-Dihydroxypropyl nonadecanoate	0.42 ^a^	ND ^b^	ND ^b^	4 ^a^	ND ^b^	ND ^b^
55.14	2,3-Dihydroxypropyl icosanoate	0.88 ^a^	0.33 ^c^	0.43 ^b^	5 ^a^	2 ^b^	1 ^c^
59.33	2,3-Dihydroxypropyl docosanoate	0.80 ^a^	0.20 ^b^	ND ^c^	4 ^a^	1 ^b^	ND ^c^
	Others	2.32 ^a^	0.90 ^c^	1.89 ^b^	15 ^a^	5 ^b^	6 ^b^
14.20	Glycerol	0.77 ^a^	0.81 ^a^	0.45 ^b^	6 ^a^	4 ^b^	1 ^c^
38.21	(9E)-Octadec-9-enoic acid ethyl ester	ND ^b^	0.09 ^a^	ND ^b^	ND ^b^	1 ^a^	ND ^b^
57.25	α-Tocopherol	1.55 ^a^	traces ^b^	1.44 ^a^	9 ^a^	traces ^c^	4 ^b^
	Total	234.19 ^a^	170.52 ^b^	172.02 ^b^	1363 ^a^	783 ^b^	446 ^c^

* Each value represents the mean of six aliquots from three extracts of each sample (standard deviation lower than 5%). Abbreviations: ND, not detected; RT, retention time; var.: variety. Tukey’s HSD test; difference in the letter in a line indicates a significant difference at *p* ≤ 0.05 between the varieties.

**Table 3:** Minimal inhibitory Concentrations of *Opuntia ficus-indica* root lipophilics expressed in (µg mL^−1^).

	Green var.	Orange var.	Red var.
Gram-negative strains			
*E. coli* ATCC 25922	550.00 ± 0.00 ^a^	566.67 ± 5.77 ^b^	606.00 ± 5.48 ^c^
*P. aeruginosa* ATCC 27853	777.50 ± 5.00 ^a^	826.00 ± 5.48 ^b^	947.50 ± 5.00 ^c^
Gram positive strains			
MRSA MU45 (Mec C)	263.33 ± 5.77 ^a^	316.67 ± 5.77 ^b^	343.33 ± 5.77 ^c^
*S. aureus* ATCC 29213	253.33 ± 5.77 ^a^	296.67 ± 5.77 ^b^	303.33 ± 5.77 ^b^
*B. cereus* ATCC 10876	76.67 ± 5.77 ^a^	83.33 ± 5.77 ^a^	86.67 ± 5.77 ^a^

Difference in the superscripts in a line indicates a significant difference between varieties regarding Tukey’s HSD test (*p* ≤ 0.05).
